# Miscellany

**Published:** 1887-06

**Authors:** 


### Miscellany.

El Kellah.—Two Egyptian physicians connected with the University of Cairo, Hassan-Pacha Mahmoud and Ibrahim Moustapha, have been investigating the therapeutic and physiological properties of a plant, *el kellah*, the alkaloid of which they have denominated *kelline*. In its action it resembles the narcotic poisons, causing vomiting, paralysis of the extremities, slowing of the respiration and irregularity of the pulse. It has been successfully employed as a gargle in sore throat and diseases of the buccal mucosa. Internally it has been used in the treatment of acute rheumatism, lithiasis and cystitis. Kellah is also said to be a tonic. It is generally given in the form of a syrup or decoction.

"Is the Cholera Coming?" is the title of a paper published by Dr. J. B. Lindsley in the *Southern Practitioner*. Reasoning from the histories of previous epidemics he believes that the disease will within a few years make its appearance in North America. Cholera has heretofore never visited Europe without crossing the ocean to make this country the field of its ravages, as the epidemics of 1833, 1849, 1854, 1866 and 1873 have demonstrated. When the cholera broke out in France in 1884, startling bulletins were published daily in this country, but we are now according to the writer in much more danger than we were at that date.

Gaseous injections in the treatment of phthisis are new, and yet disadvantages have already been discovered. Dr. Calmon, in a resumé of the subject published in *L'Abeille Medical*, concludes that the formation of tubercle is not arrested, the night sweats are not relieved, and there is no reduction of temperature. Intestinal disturbances are sometimes produced, and digestion often disturbed. He admits that calm sleep sometimes follows the use of the injection, but he attributes this to the action of the carbonic acid gas. He believes that this treatment may be advantageously used in certain cases as a palliative, but not as a cure.

---

A DEATH CERTIFICATE.—All applications for free burial in this city must be accompanied by a death certificate, written and signed by the attending physician and indorsed by the City Physician having charge of the ward in which the deceased lived. The following is a copy of a certificate which was presented the other day to the physician of the Second Ward for his indorsement:

"I sertify that Alice Pain's two twins is ded they was stillbornd."

The indorsement reads as follows: "Correct, except orthography and grammar. E. v. G., Phys. 2d Ward."

—*Southern Practitioner.*

---

THE cholera epidemic is about ended in Argentine Republic, but cases have appeared in Bolivia and Ecuador. It seems to be traveling slowly northward on the Pacific Coast. The following are the statistics of mortality in Buenos Ayres from November, 1886, to March, 1887: November, 31; December, 487; January, 355; February, 221. Total deaths, 1094. In Parana there were 990 cases and 250 deaths; in Rosario there were 154 deaths.

---

A FRENCH physician, Duchaussen, sends a communication to the *Societe Med. Practique* describing some of the effects upon health produced by the recent earthquake in the Riviera. Among others, three cases of metrorrhagia were produced; in one case the woman had not menstruated for four years. Earthquakes may be considered the latest and most powerful emmenagogue.

---

DR. HENRY WILE, Professor of Dermatology in the Atlanta Medical College and author of some of the articles in Wood's Hand-book of the Medical Sciences, died on the train between Chicago and Rochester, in April. Dr. Wile, though a young man, had attained a high rank in his profession. The cause of his death was phthisis.

---

BECHTEREW has made numerous experiments on birds, rats, dogs, etc., to determine the functions of the optic thalami, and concludes that these organs preside over the muscles in the eye which express the passions and feelings. Observations on patients with disease of the optic thalami corroborates these views.

---

GRAWITZ, Virchow's assistant, states that one-third of the cases of so-called muscular rheumatism which have been examined *post mortem* reveal the presence of *trichinae spirales* in the muscles. In many cases the parasites have been present in the muscles for years.

---

IN ancient times there were no Jewish physicians. Medical science was in the hands of foreigners in Jerusalem, and it was considered sacrilegious for an Israelite to employ them. Now it is reported that there are 900 Hebrew doctors in New York City.

---

Two physicians of St. Louis entertained their *confreres* at a meeting of the City Medical Society with a combat a la Sullivan. They were separated at the end of the first round. The difficulty arose from professional misunderstandings.

---

IN Russia there are annually 30 suicides per million inhabitants. In London there were 87 per million, in Berlin 170 per million, while Paris leads the world in this respect with 206 suicides per million.

---

REBUTEL has found that sulphurate of atropine is an almost certain cure for sea sickness. The method of administration is to give two or three hundredths of a grain subcutaneously every eight hours.

---

THE Russian Government has recently prohibited the importation of patent medicines. A list comprising some eight hundred of these articles has been published.

---

DR. J. S. JEWELL, the eminent neurologist of Chicago, is dead.

THE furniture, etc., of the Homœopathic Mutual Life Insurance Company has been attached by the sheriff.

---

THE Buffalo Medical and Surgical Association has taken new quarters in the Buffalo Library building.

---

THE United States Government has appropriated $10,000 for the use of the Ninth International Medical Congress.

---

IT is more necessary for the doctor to have a carriage than to cure his patient.—*Balzac*.

